# Effects of Unilateral Maxillary Premolar Extraction on Smile Aesthetics: A Retrospective Study -

**DOI:** 10.31661/gmj.v14iSP1.4002

**Published:** 2025-12-15

**Authors:** Seyed Moahammad Reza Safavi, Anahita Dehghani Soltani, Saeed Reza Motamedian, Alireza Akbarzadeh Baghban, Samin Ghaffari

**Affiliations:** ^1^ Department of Orthodontics, Dentofacial Deformities Research Center, Research Institute for Dental Sciences, Shahid Beheshti University of Medical Sciences, Tehran, Iran; ^2^ Department of Orthodontics, School of Dentistry, Shahid Beheshti University of Medical Sciences, Tehran, Iran; ^3^ Proteomics Research Center, Department of Biostatistics, School of Allied Medical Sciences, Shahid Beheshti University of Medical Sciences, Tehran, Iran

**Keywords:** Unilateral Premolar Extraction, Dental Midline Deviation, Smile Arc, Aesthetic

## Abstract

**Background:**

Unilateral maxillary premolar extraction (UMPE) has been recommended for the orthodontic treatment of specific dental asymmetries. This study has aimed to compare the extraction side and non-extraction side within the same patient to assess the impact of UMPE on specific dental and aesthetic outcomes.

**Materials and Methods:**

In this retrospective investigation at department of orthodontics, school of Dentistry, Shahid Beheshti University of Medical Sciences (SBMU), post treatment documents of 40 patients, who underwent UMPE in their completed orthodontic treatments, were selected. Upper dental midline, smile arc and number of visible teeth in final smile photographs was assessed. Evaluations were analyzed using SPSS 18.

**Results:**

Analyses showed a positional deviation of upper dental midline in 90% of patients by average of 1.0±0.5 mm, the angular deviation of dental midline in 87.5% of patients toward the extraction side by average of 0.83°±0.27° and an elevated smile arc for 0.43 mm in extraction side. Moreover, the evaluations showed a mean of 5 and 4.52 visible teeth in the non-extraction and the extraction sides, respectively.

**Conclusion:**

The current study showed that in the treatment with UMPE the smile indices can be end very close to absolute symmetry and the asymmetries that still exists are negligible and will not affect the aesthetic results of the treatment.

## Introduction

One of the primary goals of orthodontic treatment is to obtain an attractive and balanced smile [1][2], which is closely linked to facial esthetics [3]. To elaborate, numerous factors influence our perception of smile attractiveness, and one critical factor is the symmetry of smile indices, which is widely regarded as significant [4]. Asymmetry can manifest in various smile indices, such as the dental midline, smile arc, and the number of visible teeth [5]. Importantly, proper treatment of asymmetry relies heavily on a precise diagnosis of its etiology, which may stem from dental, skeletal, or soft tissue discrepancies [6]. Dental asymmetry and dental midline deviation are more common in Class II patients, particularly in subdivision cases [7]. To address these issues, various orthodontic treatments have been proposed for managing dental asymmetric malocclusions, including unilateral molar distalization, unilateral bimaxillary elastics, and four-premolar extraction [6][8][9]. Extraction, as a component of various orthodontic treatments, can influence treatment duration, stability, resultant occlusion, and, notably, smile esthetics [10][11].

Specifically, unilateral extraction of a maxillary premolar is described as an effective approach to correct moderate to severe asymmetries [6] and is considered advantageous for orthodontic patients because it involves less tooth structure removal. Furthermore, with appropriate case selection, patients may benefit from shorter orthodontic treatment durations [12].

Unilateral extractions are generally preferred when occlusal asymmetries are severe, to an extent that they cannot be corrected solely through asymmetric mechanics [13]. In such cases, unilateral maxillary premolar extraction may be prescribed for patients with asymmetric crowding caused by factors such as a mesially migrated first molar or dental midline deviation to the opposite side. Moreover, it offers several additional benefits, including simplified biomechanics, reduced treatment time, preservation of molar occlusion, and correction of midline deviation without creating a canted occlusal plane [13]. However, some studies have suggested that asymmetric extraction may not fully resolve arch asymmetries, potentially leading to significant residual asymmetry [14][15][16]. As a result, this could impact the characteristics of smile esthetics [17].

Conversely, other studies have concluded that there is no consistent relationship between different patterns of premolar extraction and smile esthetics [18][19][20].

Given the conflicting findings, there have been relatively few studies evaluating the effects of unilateral maxillary premolar extraction on smile indices, which could be pivotal in prioritizing this treatment approach. Therefore, the aim of this study was to assess the effects of asymmetric upper premolar extraction on residual asymmetries in smile indices, taking into account predetermined diagnostic thresholds.

## Methods and Materials

In this retrospective study, a pool of 631 patient records spanning 2015 to 2020, who had undergone unilateral upper premolar extraction and orthodontic were assessed. Post-treatment photographs of patients were selected based on specific inclusion and exclusion criteria [21]. These criteria included individuals who had undergone unilateral upper premolar extraction, completed their orthodontic treatment, and had complete treatment records, such as dental casts and adequate post-treatment frontal smile photographs, with no congenital missing teeth prior to treatment. Conversely, patients were excluded if they presented with craniofacial syndromes, cleft lip or palate, soft tissue asymmetry (including the lower lip), anterior teeth size asymmetry, or a significant Bolton discrepancy of ≥1.5 mm. Complete data and photographics were available for 40 eligible patients. Notably, these patients had completed their orthodontic treatment at the Department of Orthodontics, School of Dentistry, Shahid Beheshti University of Medical Sciences (SBMU). Furthermore, the study protocol was approved by the ethical committee of SBMU (Code: IR.SBMU.DRC.REC.1398.106). Each patient data was grouped into extraction and non-extraction categories based on whether their dental midline deviation occurred on the side of the unilateral upper premolar extraction or not.

### Outcome Measures

To evaluate the outcomes, the following information was obtained from the patients’ records: age, gender, final smile photographs, and final study cast of the upper arch. Specifically, the evaluations involved measurements taken from post-treatment smile photographs.

Photographs of posed smiles were utilized for the current study. To ensure consistency, the photos were modified using Adobe Photoshop software (Version: 19.0 x64 2017) after numbering. To minimize the effects of confounding factors, a 5×10 cm template was applied to standardize their size. Additionally, the facial midline was aligned with the template’s midline as well as the true vertical line. Moreover, the smile midline was defined by the template’s midline [22]. Finally, the photographs were saved in JPEG format.

**Figure-1:**
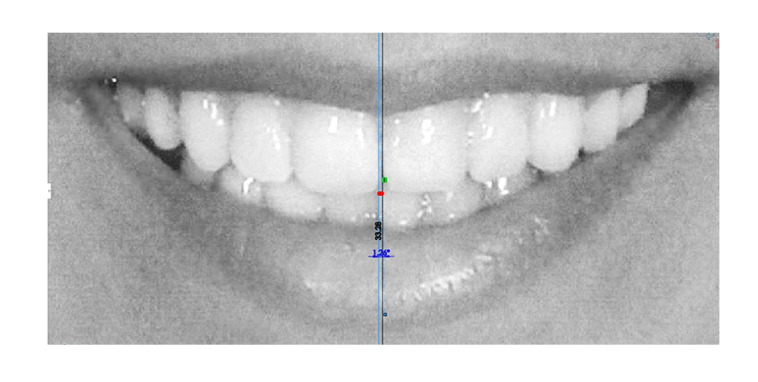
Figure 1. Evaluation of upper dental midline relative to template (face) midline

A tactile line at the contact area of the upper central incisors was designated as the upper dental midline. After magnifying the photo, its position relative to the smile midline (the template midline) was measured in tenths of a millimeter using Solidworks® software . Furthermore, the angular deviation between the upper dental midline and the smile midline was measured in tenths of a degree using the same software (Figure-1).

**Figure-2:**
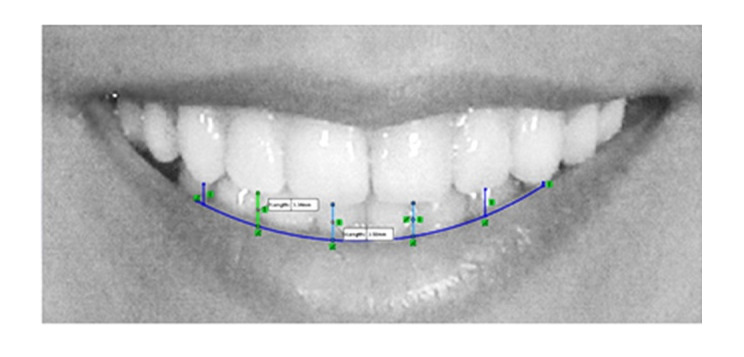
Figure 2. Evaluation of smile arc relative to the upper border of lower lip

To assess the smile arc, a line was drawn tangent to the upper border of the lower lip, and the distance between the upper anterior teeth and this line was measured along a perpendicular line drawn from the midpoint of the incisal edge of the incisors and the tip of the canine cusps on both sides [23] (Figure-2). Additionally, the number of visible teeth during smiling on each side was counted.

### Calibration, Validity, and Reliability of Measures

For calibration purposes, the mesiodistal width of the upper right central incisor was measured using a digital caliper (Insize Digital Caliper Series 1108) on the relevant stone cast and then matched with the modified photographs in Solidworks® software (3DEXPERIENCE Company, UK). Importantly, all distances were measured in tenths of a millimeter.

To ensure validity, measurements were performed by a postgraduate student of orthodontics under the supervision of an assigned professor in the corresponding department. To assess intra-examiner reliability, the evaluation of photographs was repeated for ten randomly selected specimens after a two-week interval, and measurement error analyses were conducted. The results showed that the reliability assessment indicated an intra-class correlation coefficient (ICC) of above 75% for all measurements, demonstrating high reliability based on the Rosner classification [24].

### Calibration, Validity and Reliability of Measures

For calibration of photographs, the mesiodistal width of upper right central incisor was measured by a digital caliper (Insize Digital Caliper Series 1108) on relevant stone cast and then, was matched with the modified photographs in Solidworks® software (3DEXPERIENCE Company, UK). All distances were measured in tenth of a millimeter.

In order to preserve validity, the measurements were exerted by a postgraduate student of orthodontics under the supervision of an assigned professor in the corresponding department. So as to assess the intra-examiner reliability, the evaluation of photographs was repeated for ten randomly selected specimens after two weeks and the measurement error analyses were exerted. Reliability assessment indicated an intra-class correlation coefficient (ICC) of above 75% in all measurements, showing high reliability based on Rosner classification [1].

### Statistical Analysis

Measurements were performed in all specimens. An expert, who was blind to the treatment, performed all statistical analyses. The resultant data were analyzed using SPSS software version 18.0 (IBM Corp, Chicago, IL). The significance level was set at P≤ 0.05.

## Results

**Table:** Table1. Characteristics of study participant

**Metric**	**Non-Extraction**(n=4)	**Extraction** (n=36)	**P-value**
**Dental Midline Deviation (mm)**, Mean ± SD (Min - Max)	0.3 ± 0.1 (0.2 - 0.4)	1.0 ± 0.5 (0.3 - 2.6)	0.001
**Midline Angular Deviation (°)**, Mean ± SD (Min - Max)	0.8 ± 0.2 (0.3 - 1.1)	1.4 ± 1.0 (0.2 - 3.6)	0.003
**Visible Tooth Count** (n), Mean ± SD (Min - Max)	5.0 ± 0.76 (4 - 6)	4.5 ± 0.66 (3 - 6)	<0.001

**Table:** Table2. The Distance between Inicisal Edge of Anterior Teeth and Lower Lip

**Tooth**	**Two-side distance** **difference**	**T-value**	**P-value**
**Central incisor **	0.09	-2.182	<0.036
**Lateral incisor **	0.61	-7.642	<0.001
**Canine **	0.83	-6.933	<0.001
**height of smile arch ** *	0.43	-6.63	<0.001

Twenty-seven female (67.5%) and thirteen male (32.5%) patients participated in this study with a mean age of 16.4 and SD of ?.

The position of upper dental midline was deviated to the extraction side with a mean of 1.0±0.5 mm in 90% of the specimens, while in 10% of cases, the midline was deviated to the non-extraction side with a mean of 0.3±0.1 mm (Table-1).

In addition, considering midline angular deviation, the angle was about 1.47°±1.01° to the extraction side in 87.5% of the specimens, however, the angular deviation of midline was about 0.83°±0.27° to the non-extraction side in 12.5% of the specimens. The range of angular deviation was 0.2° to 3.66° (Table-1).

The mean number of visible teeth was 5.02±0.73 at the non-extraction side and 4.52±0.64 at the extraction side. There was a statistically significant difference in this number between both sides (P<0.001, Table-1).

The mean distance between the incisal edge of anterior teeth and upper border of lower lip in each side of smile (height of smile arc) is shown in Table-2.

The mean distance was significantly higher at the extraction side compared with non-extraction side. Moreover, the differences in distance for central incisors was lower compared with lateral incisors/canines (central incisors< lateral incisors< canines). Both findings indicate the transverse roll (cant) of smile arc (P<0.05).

## Discussion

The findings of this study indicate that after orthodontic treatment with unilateral extraction of an upper premolar, all indicators were nearly symmetrical, with only minor asymmetries in smile indices and the dental arch.

With unilateral extraction of an upper premolar, the dental midline is closely aligned with the center of the smile, as observed in most cases assessed in this study. A slight midline deviation of approximately 2 mm can be detected more accurately by orthodontists [21][25][26][27], but laypersons may find midline deviations of up to 4 mm attractive, as noted by Kuruhan and others [13]. This is significantly higher than the measurements obtained in this study and is unlikely to impact smile aesthetics.

Additionally, the dental midline angulation was very close to a true vertical line. A slight deviation in midline angulation was observed in post-treatment patients, but it was minimal and negligible (less than 1 degree). This small deviation may result from initial asymmetries or asymmetric space closure mechanics. The variation in results suggests differences in space closure mechanics and clinicians’ skills [9][28][29].

This investigation revealed a high degree of similarity in the number of visible teeth between the extraction and non-extraction sides. Consequently, unilateral maxillary premolar extraction subtly affects the number of visible teeth, which may be influenced differently by unilateral space closure mechanics on each side of the dental arch. The visibility of teeth is important for smile aesthetics; however, the discrepancy between the two sides of the arch was minimal. According to NM Choma, maxillary premolar extraction does not significantly affect tooth visibility or the appearance of the smile [30].

After treatment with unilateral maxillary premolar extraction, asymmetry in the smile arc (cant of the smile arc) was not statistically significant (P<0.05). This minor asymmetry is not critical for smile aesthetics, as asymmetries farther from the midline are more clinically tolerable [31].

Unilateral maxillary premolar extraction is often prescribed as an alternative treatment when cost and complexity are concerns. Although this approach may result in minor asymmetries, these measurements fall below detectable thresholds. Moreover, some studies suggest that asymmetric tooth extraction can effectively correct malocclusion and midline deviation [32], shift posterior teeth asymmetrically, reduce dental movement time, simplify orthodontic mechanics [29][33][34][35][36][37], achieve functional and consistent results, and minimize treatment side effects [28][38].

Therefore, patient selection for such cases should consider the clinician’s expertise, as sufficient knowledge to diagnose and manage mechanics is essential for successful outcomes [39].

With proper case selection and space closure mechanics, Class II subdivision patients can be treated using asymmetric premolar extraction in the maxillary arch, which has been reported to correct midline deviation more effectively than four-premolar extraction [25].

These benefits can be achieved through accurate diagnosis of the etiology of primary asymmetries, such as molar relationships, midline deviation, anterior crowding, and other patient-specific characteristics [32]. Before tooth extraction, it is recommended to assess the degree of initial asymmetries and the anchorage required to achieve optimal symmetry [40]. After establishing appropriate anterior dental relationships, the remaining extraction space can be closed using temporary anchorage devices for posterior protraction [41].

However, prospective studies with larger sample sizes are needed to confirm appropriate case selection and space closure mechanics, given the initial severity of asymmetries in malocclusions.

Differences between the outcomes of this study and similar studies [6][41] may be attributed to variations in measurement accuracy, space closure mechanics, and clinicians’ skills.

The limitations of this study include the limited number of available specimens, the high variability in treatment mechanics, and the diverse initial malocclusion characteristics, which prevented the evaluation of statistical correlations between them.

## Conclusion

The outcomes of this retrospective study showed that in unilateral maxillary premolar extraction treatment the smile indices are very close to absolute symmetry and asymmetries were so trivial that they did not pass the recognizable thresholds determined in previous studies and does not seem to affect the aesthetic results of the treatment. Nonetheless, more comprehensive studies are needed to evaluate the smile aesthetic indices subjectively and the impact of different space closure mechanics on aesthetics treatment results. Although an important factor is the proposed treatment plan based on initial characteristics of the patients and appropriate space closure mechanics to preserve smile indices symmetry or correct the existing asymmetries

## Conflict of Interest

The authors declare that there are no conflicts of interest regarding the publication of this paper.
